# Supplementary material for: High Poly(ADP-Ribose) Polymerase Expression Does Relate to Poor Survival in Solid Cancers: A Systematic Review and Meta-Analysis
Source: Cancers (Basel). 2021 Nov 9;13(22):5594. doi: 10.3390/cancers13225594 (PMC8615806; doi:10.3390/cancers13225594)
Supplement: Supplementary file 1 [file cancers-13-05594-s001.zip › cancers-1406313-supplementary.pdf]

cancers

MDPI

Supplementary Data: Clinicopathological and prognostic significance of poly(ADP-ribose) expression in various solid cancers: a systematic review and meta-analysis

Citation: Thakur, N.; Yim, K.; Abdul-Ghafar, J.; Seo, K.J.; Chong, Y. High Poly(ADP-Ribose) Polymerase Expression Does Relate to Poor Survival in Solid Cancers: A Systematic Review and Meta-Analysis. Cancers **2021**, *13*, 5594. https://doi.org/10.3390/cancers13225594

Academic Editor: Robert C. Bast, Jr.

Received: 18 September 2021
Accepted: 5 November 2021
Published: 9 November 2021

Publisher's Note: MDPI stays neutral with regard to jurisdictional claims in published maps and institutional affiliations.

Copyright: © 2021 by the authors. Submitted for possible open access publication under the terms and conditions of the Creative Commons Attribution (CC BY) license (http://creativecommons.org/licenses/by/4.0/).

Index

Supplementary TableS1. Results of quality assessment

Supplementary Table S2. Scoring system of immunohistochemical staining for PARPs used in the included studies.

Supplementary Figure S1. Forest plot for studies evaluating the hazard ratio for PARPs expression and overall survival (OS), (A) univariate analysis, (B)

Supplementary Figure S2. Subgroup analysis between PARPs expression and overall survival (OS), (A) according to type of sample size, (B) source of hazard. ratio.

Supplementary Figure S3. Subgroup analysis between PARP expression and (**A)** overall survival (OS) and (**B)** progression-free survival (PFS) according to chemotherapy regimen

Supplementary Figure S4. Subgroup analysis between PARP expression and (**A)** disease-free survival (DFS) according to cancer types, and (**B)** progression-free survival (PFS) according to cancer types

Supplementary Figure S5. Forest plot for studies evaluating the hazard ratio (HR) for PARPs expression and disease frees survival (DFS) and progression free survival (PFS) (A) univariate analysis, (B) multivariate analysis in solid cancer patients.

Supplementary Figure S6. Forest plot evaluating the subgroup analysis between PARPs expression and (**A)** disease-free survival (DFS) and progression-free survival (PFS) according to ethnicity (Asian vs. Caucasian) **(B)** DFS and PFS of direct and indirect methods (pooled HRs versus K-M curve data extraction)

Supplementary Figure S7. Forest plot of the association between PARPs expression and clinicopathological characteristics. (A) Age, (B) gender, (C) tumour size, (D) histological grade, (E) tumour stage, and (F) lymph node metastasis.

Supplementary Figure S8. Forest plot of the association between PARPs expression and clinicopathological characteristics. (A) TNM stage, (B) lymph vascular invasion, (C) Ki-67, (D) BRCA1, and (E) BRCA2

Supplementary Figure S9. Funnel Plot (A) overall survival, (C) disease-free survival (E) progression-free survival and trim and fill funnel plot (B) overall survival, (D) disease-free survival (F) progression-free survival.

Supplementary Table S1. Results of quality assessment

First author, year	Selection[1]				Comparability[2]	Outcome[3]			Total Score
	Exposed Cohort ★	non-exposed cohort ★	Ascertainment of exposure ★	Outcome of the study was not presented at the start of study★	Control for important factor or additional factor ★★	Outcome Assessment ★	Adequate follow-up★	Loss to follow-up★	
Gonclaves, 2011 [1]	★	★	★		★★	★	★	★	8
Minckwitz, 2011 [2]	★	★	★		★★	★	★	★	8
Rojo, 2012 [3]	★	★	★		★★	★	★	★	8
Donizy,2014 [4]	★	★	★		★	★	★		7
Aiad, 2015 [5]	★	★	★		★★	★	★		7
Green, 2015 [6]	★	★	★		★★	★	★		7
Park, 2015 [7]	★	★	★		★★	★	★	★	8
Zhai, 2015 [8]	★	★	★		★★	★	★	★	8
Mazzotta, 2016 [9]	★	★	★		★★	★	★	★	8
Deng, 2017 [10]	★	★	★		★★	★	★	★	8
Mangia, 2017 [11]	★	★	★		★★	★	★	★	8
Siraj, 2018 [12]	★	★	★		★★	★	★	★	8
Song, 2017 [13]	★	★	★		★★	★	★	★	8
Brustmann, 2007 [14]	★	★	★		★★	★	★		7
Barnett, 2010 [15]	★	★	★		★★	★	★		7
Gan, 2013 [16]	★	★	★		★★	★	★		7
Ali, 2019 [17]	★	★	★		★★	★	★		7
Molnar, 2020 [18]	★	★	★		★★	★	★	★	8
Molnar, 2021 [19]	★	★	★		★★	★	★	★	8
Kim, 2014 [20]	★	★	★		★★	★	★	★	8
Xie, 2014 [21]	★	★	★		★★	★	★		7

Michels, 2015 [22]	★	★	★		★★	★	★	★	8
Lin, 2016 [23]	★	★	★		★★	★	★		7
Yu, 2019 [24]	★	★	★		★★	★	★		7
Li, 2016 [25]	★	★	★		★	★	★		7
Murnyák, 2017 [26]	★	★	★		★★	★	★		7
Yamamoto, 2017 [27]	★	★	★		★★	★	★	★	8
Klauschen, 2012 [28]	★	★	★		★★	★	★		7
Donizy, 2020 [29]	★	★	★		★★	★	★	★	8
Kim, 2016 [30]	★	★	★		★★	★	★	★	8
Liu, 2016 [31]	★	★	★		★★	★	★	★	8

1 “Selection” part includes representativeness of cases, selection of controls, exposure ascertainment, and no death when investigation started.

2 “Comparability” part includes comparison between each group and addition factors comparable on confounders.

3 “Outcome” part includes outcome assessment, adequate follow-up, and loss to follow-up rate.

Supplementary Table S2. Scoring system of immunohistochemical staining for PARPs used in the included studie

Scoring system	Study/Year	Organs	Staining Percentage (SP)	Staining Intensity (SI)	Scoring formula	Prognostic groups	Score range	cut off value for statistical analysis
H score	Green, 2015 [6]	Breast	0-100%		((SP of 1+) x 1)+ ((SP of 2+) x 2)+ ((SP of 3+) x 3)	Low: 0-10<br>High: 11-300	0-300	10
	Aiad (1)*, 2015 [5]					Low: 0-70<br>High: 71-300		70
	Aiad (2)*, 2015 [5]					Low: 0-10<br>High: 11-300		10
	Song, 2017 [13]					Low: 0-57.5		57.5

*: Aiad (1): target cytoplasmic PARP, Aiad (2): target nuclear PARP, Gan (1), Target Nuclear PARP-1, and Gan (2): target nuclear cleaved PARP-1.

						High: 57.5-300			§: Not clearly stated but presented in figure.
	Gan (1)*, 2013 [16]					Low: 0-180<br>High: 181-300		180	
	Gan (2)*, 2013 [16]	Ovary		0: No staining<br>1: Weak<br>2: Intermediate<br>3: Strong staining		Low: 0-75<br>High: 76-300		75	
	Ali, 2019 [17]					Low: 0-80<br>High: 81-300		80	
	Michels, 2015 [22]	Lung				Low: 0-145<br>High:146-300		145§	
	Liu, 2016 [31]	Liver				Low: 0-175<br>High:176-300		175	
	Donizy, 2020 [29]	Skin				Low 0-200<br>High 201-300		200	
Immunoreactivity score (IRS)	Brustmann, 2007 [14]	Ovary			SP x SI	Negative: 0-1<br>Low: 2-6<br>High 8-12	0-12	8	
	Barnett, 2010 [15]		1: 1-10%,<br>2: 11-50%<br>3: 51-80%<br>4: 81-100%			Low: 0-8<br>High: 8-12			
	Minckwitz, 2011 [2]	Breast				Negative: 0-2<br>Intermediate: 3-4,<br>High: 6-12		6	
	Klauschen, 2012 [28]	Pancreas				Negative: 0-2<br>Positive: 3-12		3	
	Xie, 2014 [21]	Lung	1: 1-10%<br>2: 11-50%<br>3: 51-75%<br>4: 75-100%			Negative: 0-4<br>Positive: 4-12		4	
	Donizy, 2014 [4]	Breast				Low: 0-6<br>High: 6-12		6	

Quickscore<br>(QS)	Zhai, 2015 [8]<br>Mazzotta, 2016 [9]<br>Deng, 2017 [4]<br>Mangia, 2017 [11]<br>Siraj, 2018 [12]	Breast	1: 1–4%<br>2: 5–19%;<br>3: 20–39%<br>4: 40–59%<br>5: 60–79%<br>6: 80–100%	0: No staining<br>1: Weak<br>2: Intermediate<br>3: Strong staining	SP x SI	Low: 0-9<br>High: 10-18	0-18	10
SI-based scoring	Lin, 2016* [23]<br>Yamamoto, 2017 [27]	Liver<br>Stomach	-		SI	Low: 0-1<br>High: 2-3	0-3	2
	Molnár, 2020 [18]<br>Molnár, 2021[19]	Ovary				Negative: 0<br>Positive: 1-3		1
Allred score	Park, 2015 [7]	Breast	1: 1%,<br>2: 2-10%,<br>3: 11-33%,<br>4: 34-66%<br>5: 67-100%		(SP + SI) of<br>2 TMA cores	Low: 0-12<br>High: 13-16	0-16	13
	Kim, 2016 [30]	Soft tissue				Low: 0-10<br>High: 11-16-	0-16	10
Summation based<br>scoring	Li, 2016 [25]	Bone	0: none;<br>1: 1-25%<br>2: 26–50%;<br>3: 51-100%.		SP + SI	Low: 0-2<br>High: 3-6	0-6	3
Computer based<br>scoring	Rojo, 2012 [3]	Breast	-		-	Optical density of<br>29 to 133 094.	29-133.094-	NA<br>(29 to 133<br>094).
SP-based scoring	Kim, 2014 [20]	Lung	0: 1-10%,<br>1: 11%-30%<br>2: 31-60%		SP	Low: 0-2<br>High: 3	0-3	3

3: 61-100%

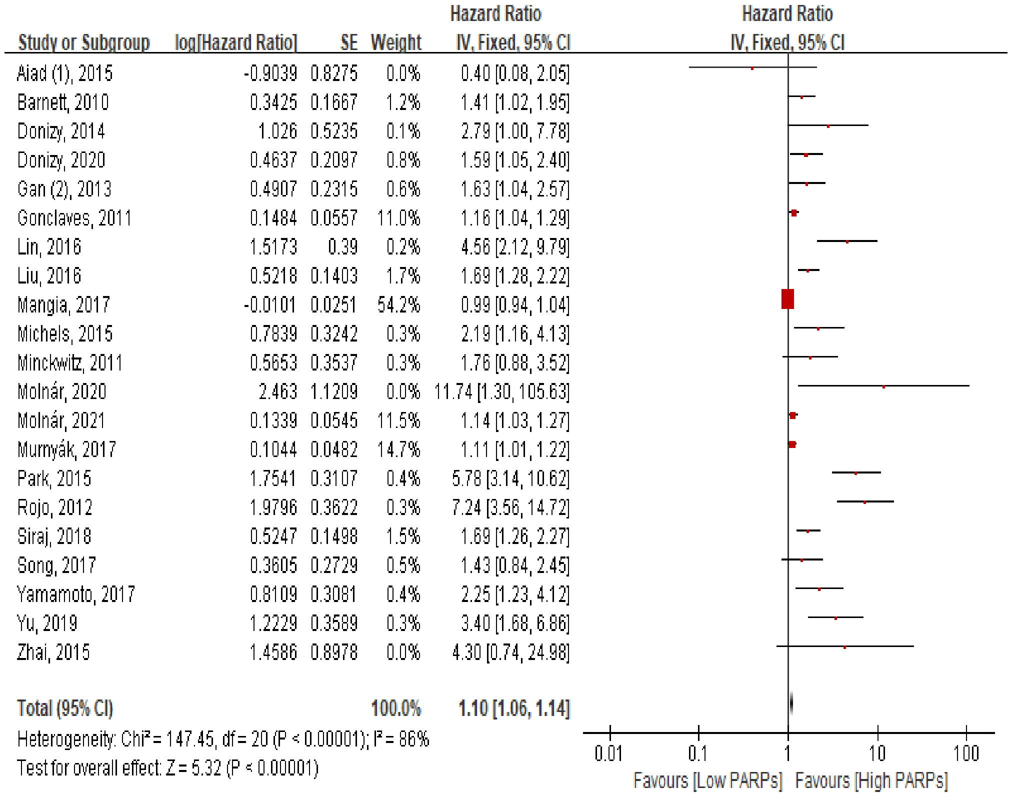

A. OS (Univariate analysis)

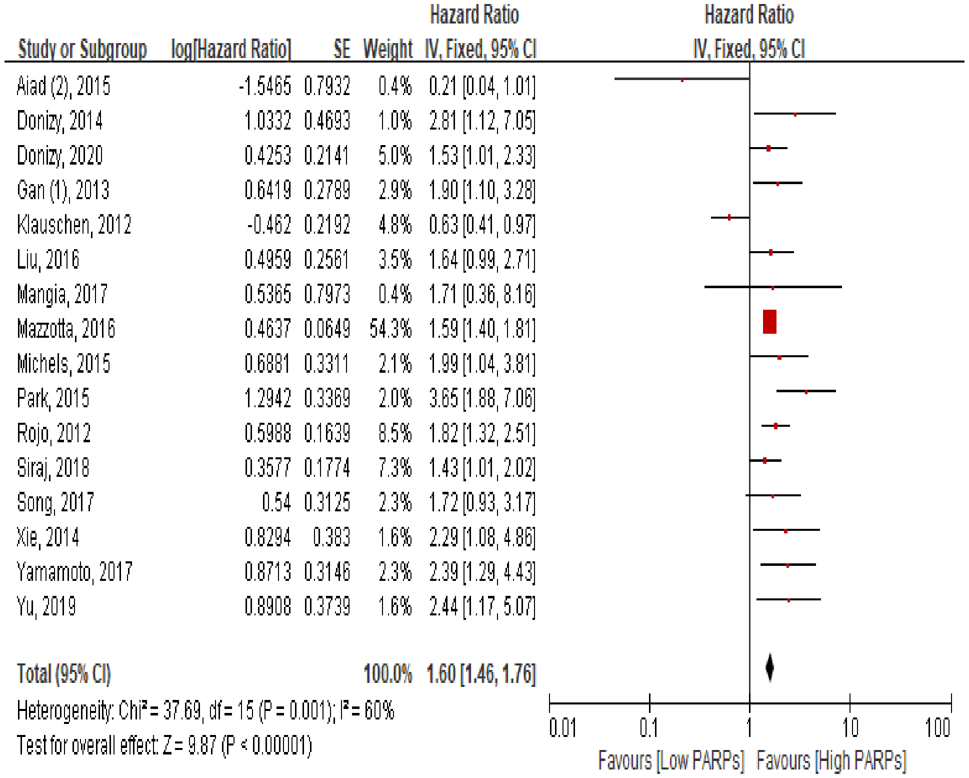

B. OS (Multivariate analysis)

Supplementary Figure S1. Forest plot for studies evaluating the hazard ratio for PARPs expression and overall survival (OS), (**A**) univariate analysis, (**B**) multivariate analysis in solid cancer patients.

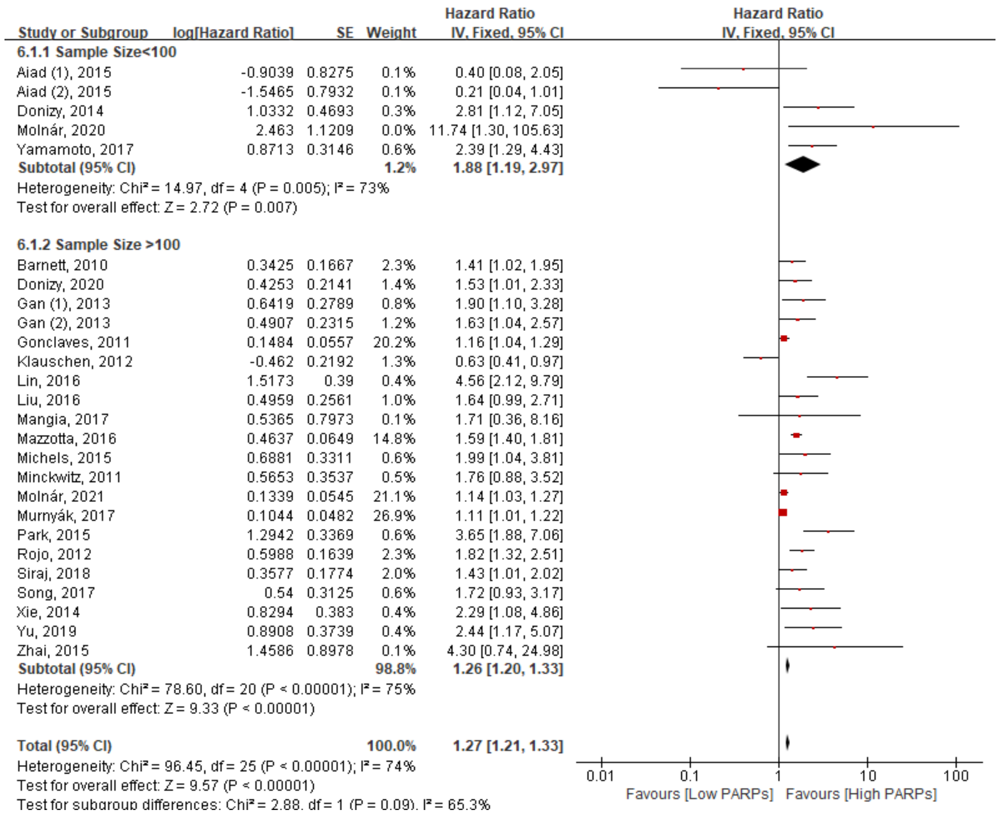

A. OS (Sample size)

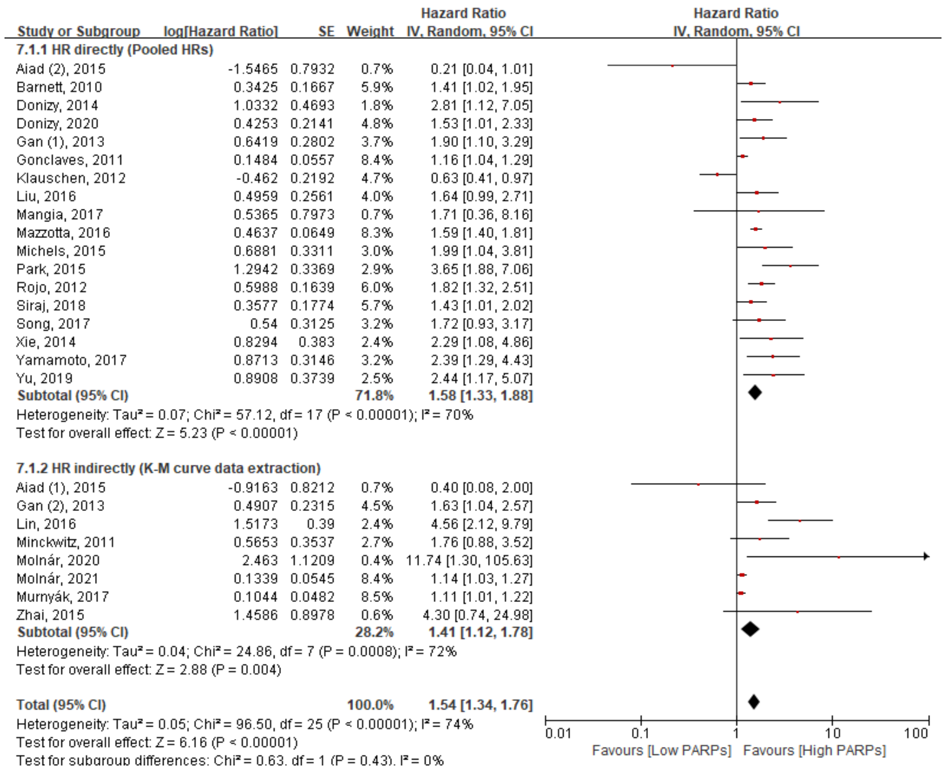

B. OS (Source of hazard. Ratio)

Supplementary Figure S2. Subgroup analysis between PARPs expression and overall survival (OS), (**A**) according to type of sample size, (**B**) source of hazard. ratio.

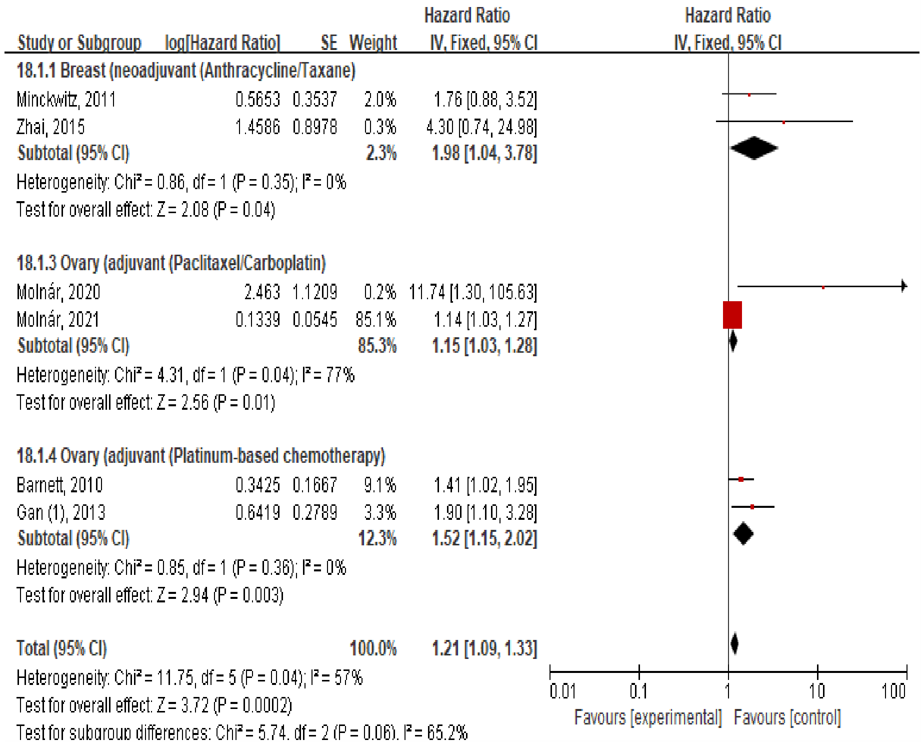

A. OS (according to chemotherapy regimen)

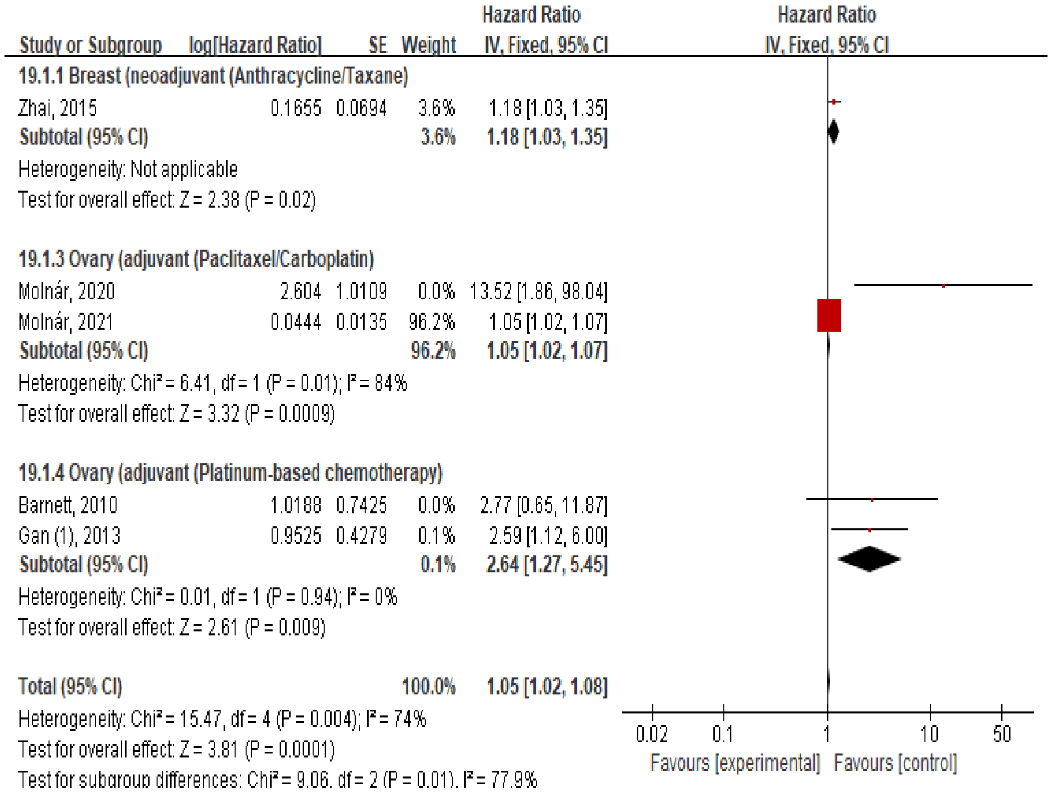

B. PFS (according to chemotherapy regimen)

Supplementary Figure 3. Subgroup analysis between PARP expression and (**A**) overall survival (OS) and (**B**) progression-free survival (PFS) according to chemotherapy regimen

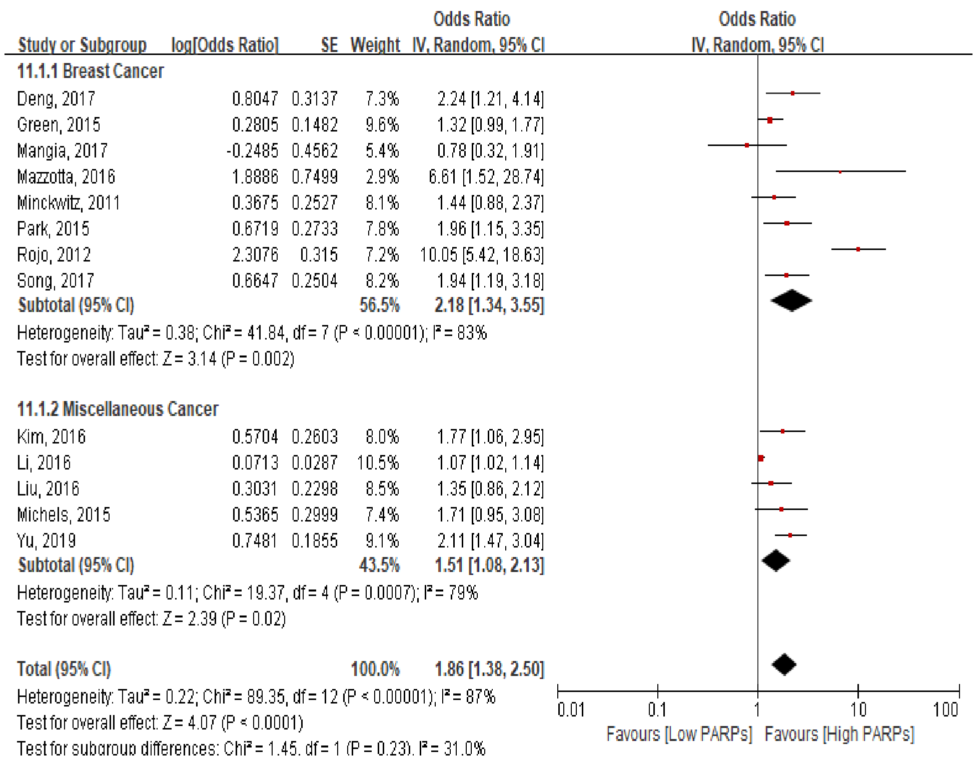

A DFS (according to cancer types)

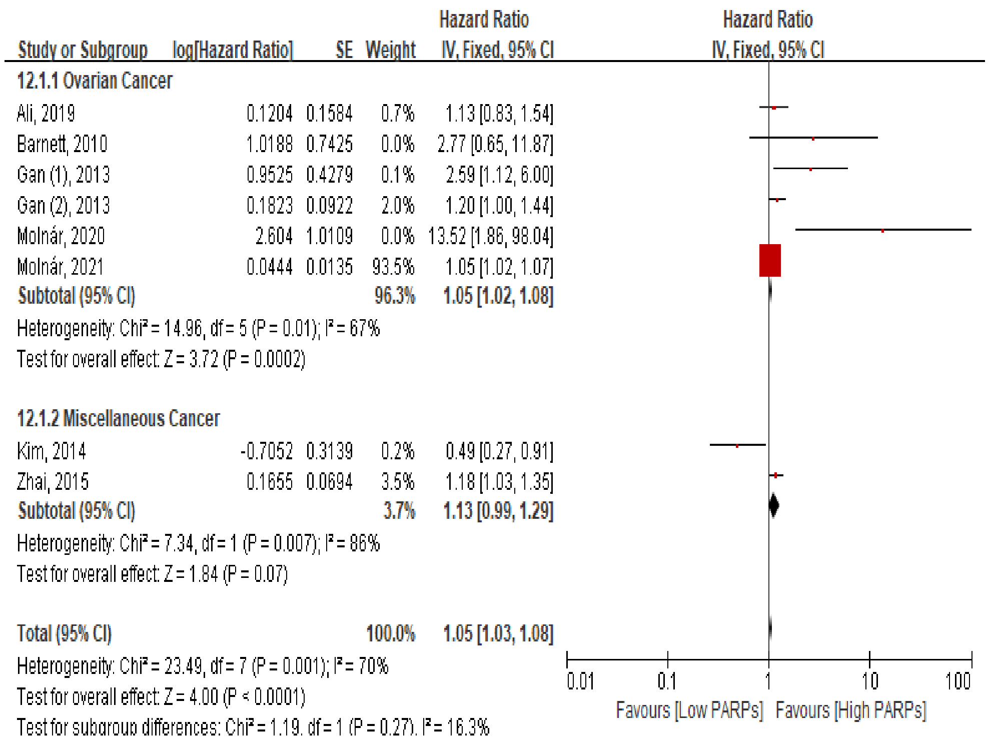

B PFS (according to cancer types)

Supplementary Figure S4. Subgroup analysis between PARP expression and (**A)** disease-free survival (DFS) according to cancer types, and (**B)** progression-free survival (PFS) according to cancer types.

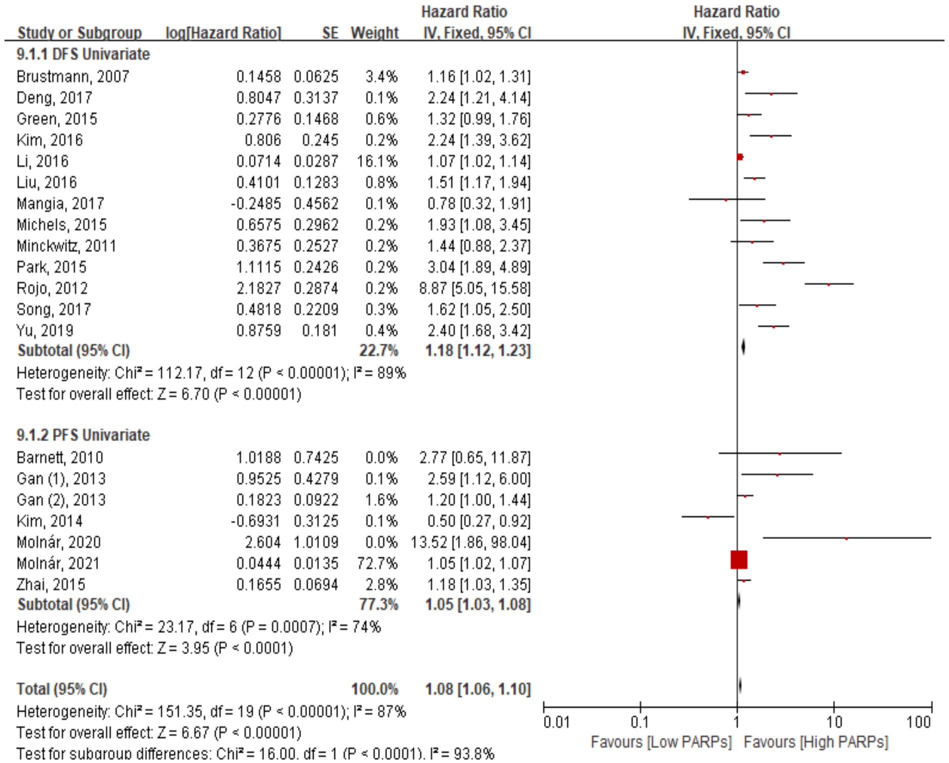

A. DFS and PFS (Univariate analysis)

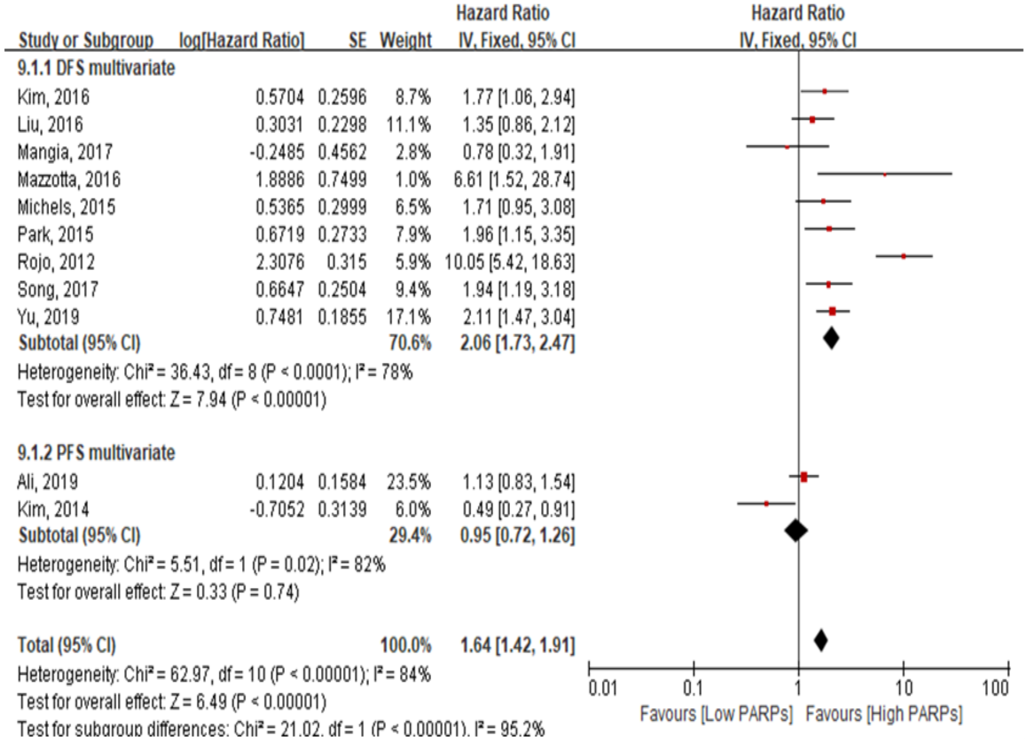

B. DFS and PFS (Multivariate analysis)

Supplementary Figure S5. Forest plot for studies evaluating the hazard ratio (HR) for PARPs expression and disease frees survival (DFS) and progression free survival (PFS) (A) univariate analysis, (B) multivariate analysis in solid cancer patients.

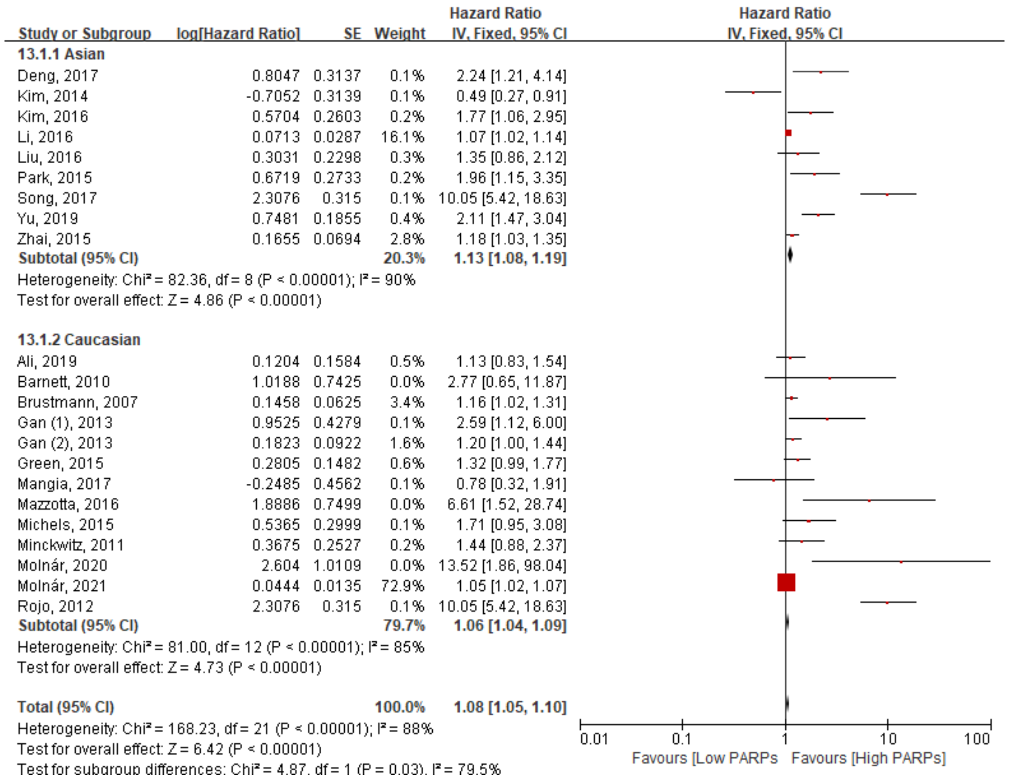

A DFS and PFS (Ethnicity)

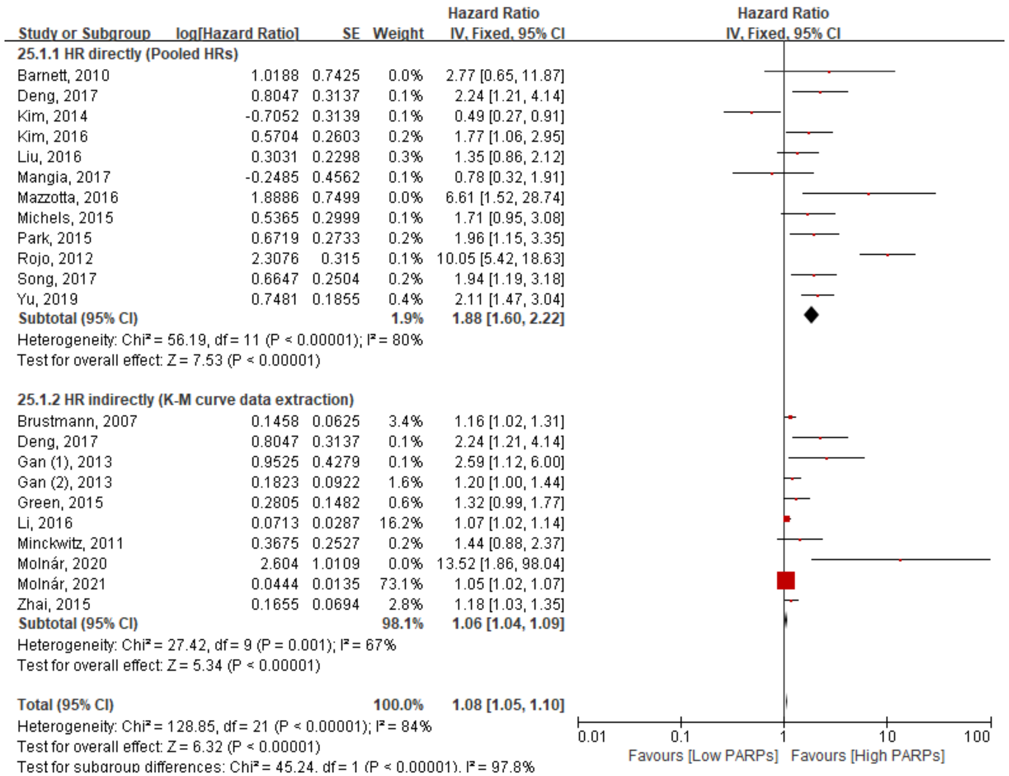

B DFS and PFS (direct vs. indirect methods)

Supplementary Figure S6 Forest plot evaluating the subgroup analysis between PARPs expression and (**A)** disease-free survival (DFS) and progression-free survival (PFS) according to ethnicity (Asian vs. Caucasian) (**B)** DFS and PFS of direct and indirect methods (pooled HRs versus K-M curve data extraction).

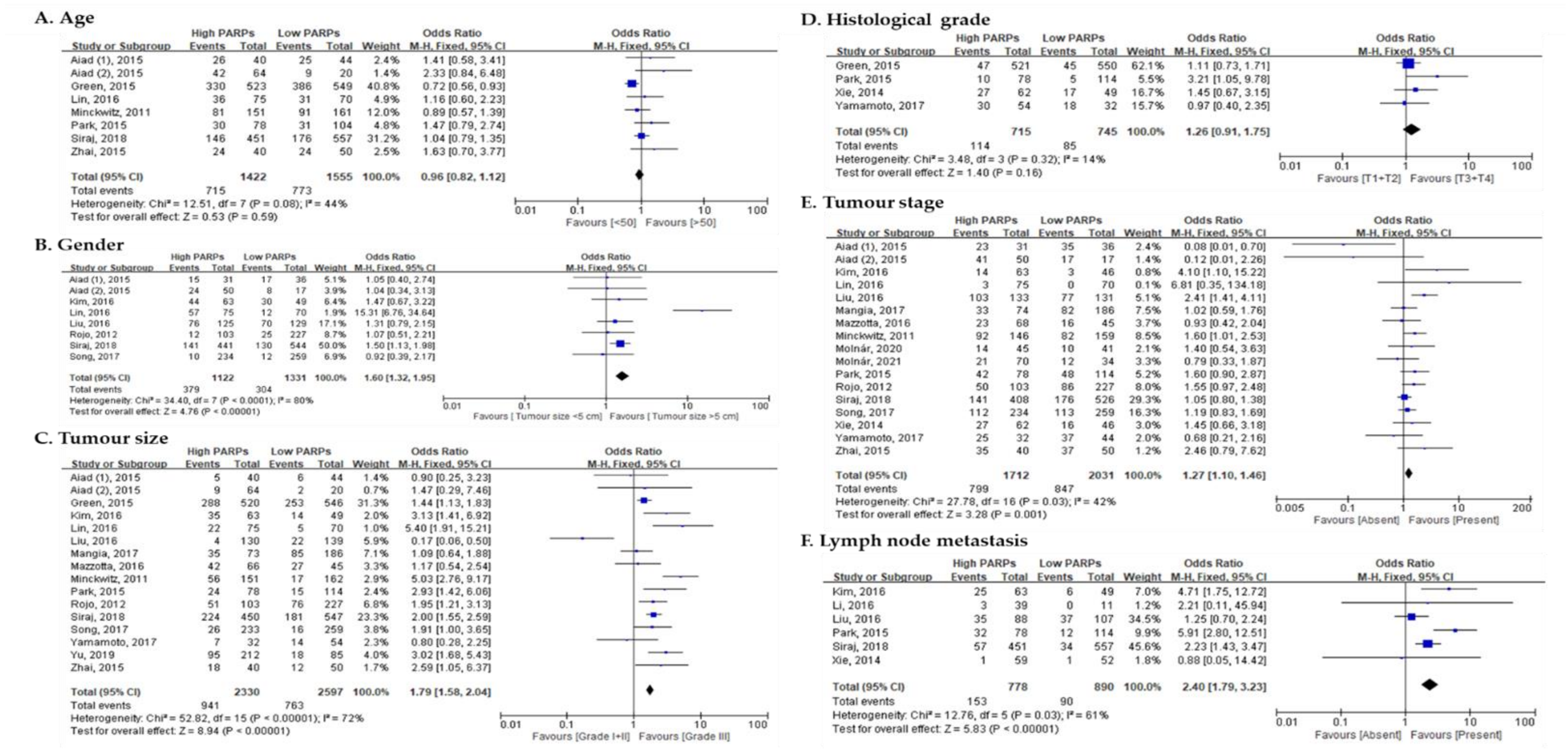

Supplementary Figure S7. Forest plot of the association between PARPs expression and clinicopathological characteristics. (A) Age, (B) gender, (C) tumour size, (D) histological grade, (E) tumour stage, and (F) lymph node metastasis.

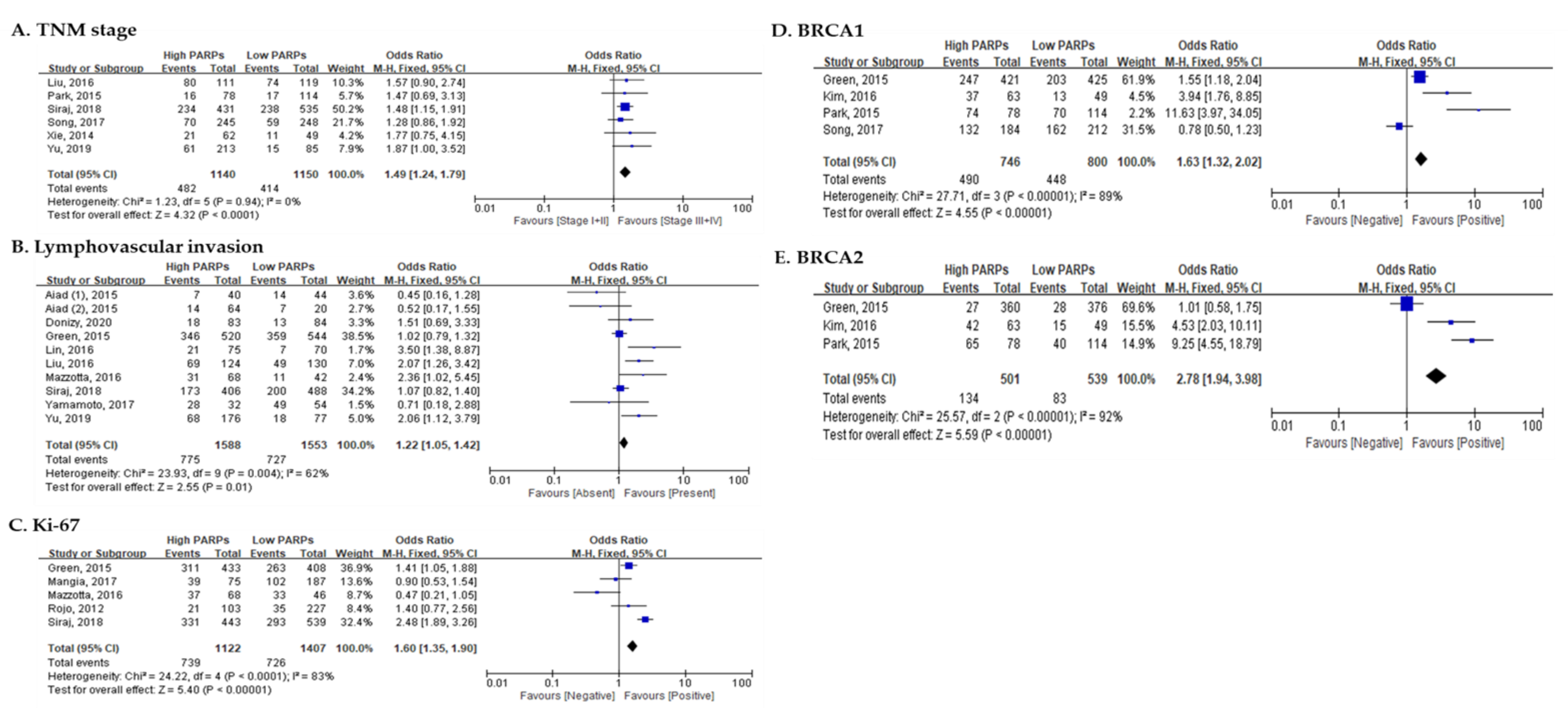

Supplementary Figure S8. Forest plot of the association between PARPs expression and clinicopathological characteristics. (A) TNM stage, (B) lymph vascular invasion, (C) Ki-67, (D) BRCA1, and (E) BRCA

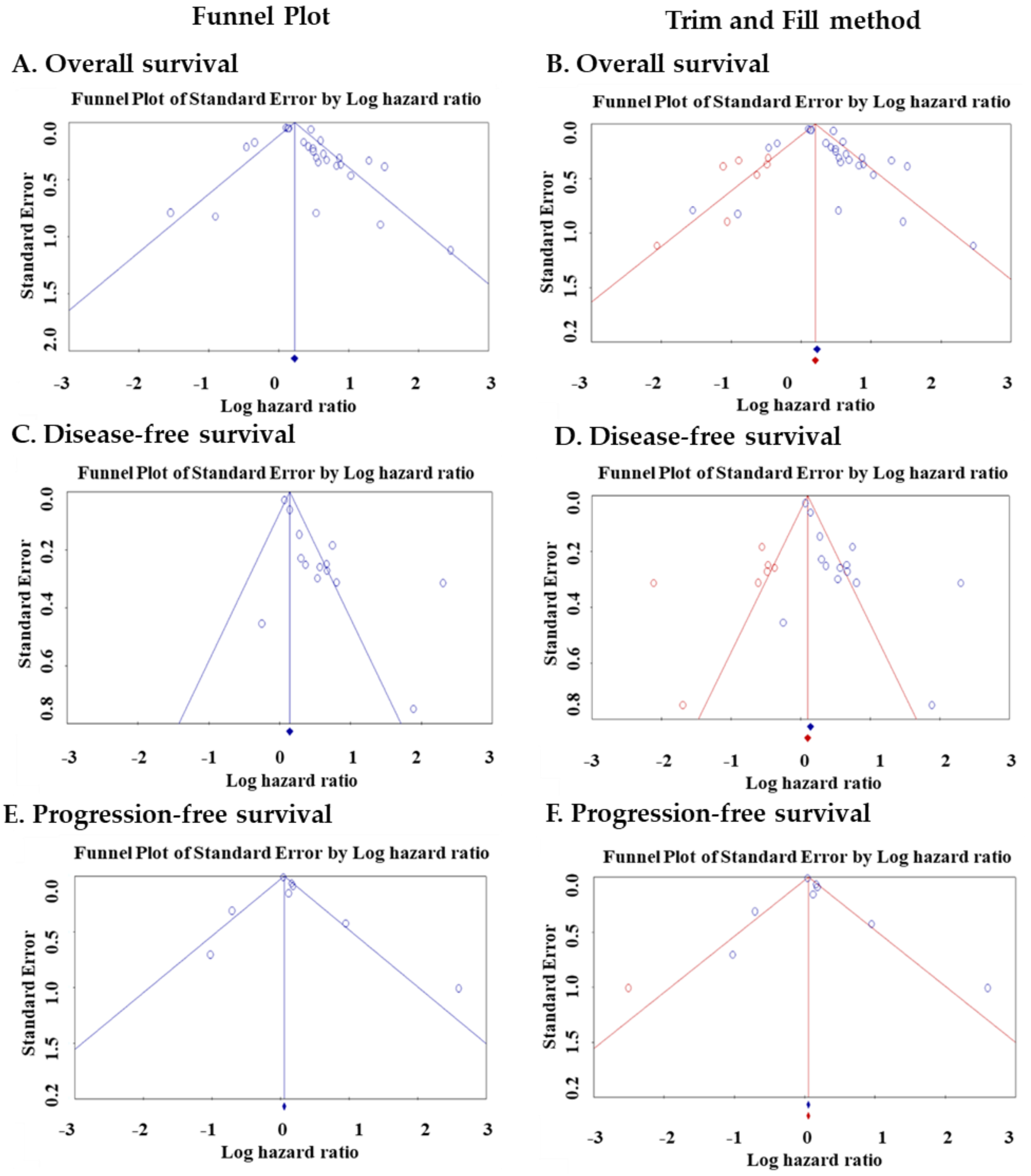

Supplementary Figure S9. Funnel Plot (A) overall survival, (C) disease-free survival (E) progression-free survival and trim and fill funnel plot (B) overall survival, (D) disease-free survival (F) progression-free survival.

References

1. Gonçalves, A.; Finetti, P.; Sabatier, R.; Gilabert, M.; Adelaide, J.; Borg, J.-P.; Chaffanet, M.; Viens, P.; Birnbaum, D.; Bertucci, F. Poly(ADP-ribose) polymerase-1 mRNA expression in human breast cancer: a meta-analysis. *Breast Cancer Research and Treatment* **2011**, *127*, 273-281.
2. von Minckwitz, G.; Müller, B.M.; Loibl, S.; Budczies, J.; Hanusch, C.; Darb-Esfahani, S.; Hilfrich, J.; Weiss, E.; Huober, J.; Blohmer, J.U.; et al. Cytoplasmic poly(adenosine diphosphate-ribose) polymerase expression is predictive and prognostic in patients with breast cancer treated with neoadjuvant chemotherapy. *J Clin Oncol* **2011**, *29*, 2150-2157.
3. Rojo, F.; García-Parra, J.; Zazo, S.; Tusquets, I.; Ferrer-Lozano, J.; Menendez, S.; Eroles, P.; Chamizo, C.; Servitja, S.; Ramírez-Merino, N.; et al. Nuclear PARP-1 protein overexpression is associated with poor overall survival in early breast cancer. *Annals of Oncology* **2012**, *23*, 1156-1164.

4. Donizy, P.; Pietrzyk, G.; Halon, A.; Kozyra, C.; Gansukh, T.; Lage, H.; Surowiak, P.; Matkowski, R. Nuclear-cytoplasmic PARP-1 expression as an unfavorable prognostic marker in lymph node-negative early breast cancer: 15-year follow-up. *Oncology Reports* **2014**, *31*, 1777-1787.
5. Aiad, H.A.; Kandil, M.A.; El-Tahmody, M.A.; Abulkheir, I.L.; Abulkasem, F.M.; Elmansori, A.A.; Aleskandarany, M.A. The prognostic and predictive significance of PARP-1 in locally advanced breast cancer of Egyptian patients receiving neoadjuvant chemotherapy. *Appl Immunohistochem Mol Morphol* **2015**, *23*, 571-579.
6. Green, A.R.; Caracappa, D.; Benhasouna, A.A.; Alshareeda, A.; Nolan, C.C.; Macmillan, R.D.; Madhusudan, S.; Ellis, I.O.; Rakha, E.A. Biological and clinical significance of PARP1 protein expression in breast cancer. *Breast Cancer Research and Treatment* **2015**, *149*, 353-362.
7. Park, S.-H.; Noh, S.J.; Kim, K.M.; Bae, J.S.; Kwon, K.S.; Jung, S.H.; Kim, J.R.; Lee, H.; Chung, M.J.; Moon, W.S.; et al. Expression of DNA Damage Response Molecules PARP1, γH2AX, BRCA1, and BRCA2 Predicts Poor Survival of Breast Carcinoma Patients. *Translational Oncology* **2015**, *8*, 239-249.
8. Zhai, L.; Li, S.; Li, X.; Li, H.; Gu, F.; Guo, X.; Liu, F.; Zhang, X.; Fu, L. The nuclear expression of poly (ADP-ribose) polymerase-1 (PARP1) in invasive primary breast tumors is associated with chemotherapy sensitivity. *Pathol Res Pract* **2015**, *211*, 130-137.
9. Mazzotta, A.; Partipilo, G.; De Summa, S.; Giotta, F.; Simone, G.; Mangia, A. Nuclear PARP1 expression and its prognostic significance in breast cancer patients. *Tumour Biol* **2016**, *37*, 6143-6153.
10. Deng, L.; Lei, Q.; Wang, Y.; Wang, Z.; Xie, G.; Zhong, X.; Wang, Y.; Chen, N.; Qiu, Y.; Pu, T. Downregulation of miR-221-3p and upregulation of its target gene PARP1 are prognostic biomarkers for triple negative breast cancer patients and associated with poor prognosis. *Oncotarget* **2017**, *8*, 108712.
11. Mangia, A.; Scarpi, E.; Partipilo, G.; Schirosi, L.; Opinto, G.; Giotta, F.; Simone, G. NHERF1 together with PARP1 and BRCA1 expression as a new potential biomarker to stratify breast cancer patients. *Oncotarget* **2017**, *8*, 65730-65742.
12. Siraj, A.K.; Pratheeshkumar, P.; Parvathareddy, S.K.; Divya, S.P.; Al-Dayel, F.; Tulbah, A.; Ajarim, D.; Al-Kuraya, K.S. Overexpression of PARP is an independent prognostic marker for poor survival in Middle Eastern breast cancer and its inhibition can be enhanced with embelin co-treatment. *Oncotarget* **2018**, *9*, 37319-37332.
13. Song, Z.; Wang, Y.; Xiao, Q.; Yu, Z.; Zhao, L.; Wu, H.; Sun, M.; Chai, Z.; Hou, P.; Geng, X.; et al. Poly(ADP-ribose) polymerase-3 overexpression is associated with poor prognosis in patients with breast cancer following chemotherapy. *Oncology letters* **2018**, *16*, 5621-5630.
14. Brustmann, H. Poly(adenosine diphosphate-ribose) polymerase expression in serous ovarian carcinoma: correlation with p53, MIB-1, and outcome. *Int J Gynecol Pathol* **2007**, *26*, 147-153.
15. Barnett, J.C.; Kondoh, E.; Whitaker, R.; Murphy, S.K.; Berchuck, A. High poly (ADP-ribose) polymerase (PARP) expression is associated with poor survival in advanced-stage serous ovarian cancer. *Gynecologic Oncology* **2009**, *112*, S107.
16. Gan, A.; Green, A.R.; Nolan, C.C.; Martin, S.; Deen, S. Poly(adenosine diphosphate-ribose) polymerase expression in BRCA-proficient ovarian high-grade serous carcinoma; association with patient survival. *Hum Pathol* **2013**, *44*, 1638-1647.
17. Ali, R.; Alabdullah, M.; Alblihy, A.; Miligy, I.; Mesquita, K.A.; Chan, S.Y.; Moseley, P.; Rakha, E.A.; Madhusudan, S. PARP1 blockade is synthetically lethal in XRCC1 deficient sporadic epithelial ovarian cancers. *Cancer Lett* **2020**, *469*, 124-133.
18. Molnár, S.; Beke, L.; Méhes, G.; Póka, R. The Prognostic Value of PARP Expression in High-Grade Epithelial Ovarian Cancer. *Pathology & Oncology Research* **2020**, *26*, 2549-2555.
19. Molnár, S.; Vida, B.; Beke, L.; Méhes, G.; Póka, R. The Prognostic Relevance of Poly (ADP-Ribose) Polymerase Expression in Ovarian Cancer Tissue of Wild Type and BRCA-Mutation Carrier Patients. Diagnostics 2021, 11, 144.
20. Kim, H.C.; Song, J.S.; Lee, J.C.; Lee, D.H.; Kim, S.W.; Lee, J.S.; Kim, W.S.; Rho, J.K.; Kim, S.Y.; Choi, C.M. Clinical significance of NQO1 polymorphism and expression of p53, SOD2, PARP1 in limited-stage small cell lung cancer. Int J Clin Exp Pathol 2014, 7, 6743-6751.
21. Xie, K.J.; He, H.E.; Sun, A.J.; Liu, X.B.; Sun, L.P.; Dong, X.J. Expression of ERCC1, MSH2 and PARP1 in non-small cell lung cancer and prognostic value in patients treated with platinum-based chemotherapy. Asian Pac J Cancer Prev 2014, 15, 2591-2596.
22. Michels, J.; Adam, J.; Goubar, A.; Obrist, F.; Damotte, D.; Robin, A.; Alifano, M.; Vitale, I.; Olaussen, K.; Girard, P. Negative prognostic value of high levels of intracellular poly (ADP-ribose) in non-small cell lung cancer. *Annals of Oncology* **2015**, *26*, 2470-2477
23. Lin, L.; Zhang, Y.D.; Chen, Z.Y.; Chen, Y.; Ren, C.P. The clinicopathological significance of miR-149 and PARP-2 in hepatocellular carcinoma and their roles in chemo/radiotherapy. *Tumour Biol* **2016**, *37*, 12339-12346.
24. Yu, B.; Ding, Y.; Liao, X.; Wang, C.; Wang, B.; Chen, X. Overexpression of PARPBP Correlates with Tumor Progression and Poor Prognosis in Hepatocellular Carcinoma. Dig Dis Sci 2019, 64, 2878-2892.
25. Li, Z.; Lv, T.; Liu, Y.; Huang, X.; Qiu, Z.; Li, J. PARP1 is a novel independent prognostic factor for the poor prognosis of chordoma. Cancer Biomark 2016, 16, 633-639.
26. Murnyák, B.; Kouhsari, M.C.; Hershkovitch, R.; Kálmán, B.; Marko-Varga, G.; Klekner, Á.; Hortobágyi, T. PARP1 expression and its correlation with survival is tumour molecular subtype dependent in glioblastoma. Oncotarget 2017, 8, 46348-46362,
27. Yamamoto, M.; Yamasaki, M.; Tsukao, Y.; Tanaka, K.; Miyazaki, Y.; Makino, T.; Takahashi, T.; Kurokawa, Y.; Nakajima, K.; Takiguchi, S.; et al. Poly (ADP-ribose) polymerase-1 inhibition decreases proliferation through G2/M arrest in esophageal squamous cell carcinoma. Oncol Lett 2017, 14, 1581-1587

28. Klauschen, F.; von Winterfeld, M.; Stenzinger, A.; Sinn, B.V.; Budczies, J.; Kamphues, C.; Bahra, M.; Wittschieber, D.; Weichert, W.; Striefler, J.; et al. High nuclear poly-(ADP-ribose)-polymerase expression is prognostic of improved survival in pancreatic cancer. Histopathology 2012, 61, 409-416.
29. Donizy, P.; Wu, C.L.; Mull, J.; Fujimoto, M.; Chłopik, A.; Peng, Y.; Shalin, S.C.; Angelica Selim, M.; Puig, S.; Fernandez-Figueras, M.T.; et al. Up-regulation of PARP1 expression significantly correlated with poor survival in mucosal melanomas. Cells 2020, 9.
30. Kim, K.M.; Moon, Y.J.; Park, S.H.; Park, H.J.; Wang, S.I.; Park, H.S.; Lee, H.; Kwon, K.S.; Moon, W.S.; Lee, D.G.; et al. Individual and Combined Expression of DNA Damage Response Molecules PARP1, γH2AX, BRCA1, and BRCA2 Predict Shorter Survival of Soft Tissue Sarcoma Patients. PLoS One 2016, 11, e0163193.
31. Liu, Y.; Zhang, Y.; Zhao, Y.; Gao, D.; Xing, J.; Liu, H. High PARP-1 expression is associated with tumor invasion and poor prognosis in gastric cancer. Oncology letters 2016, 12, 3825-3835.
